# Mesenchymal Stem Cell Therapy for Spinal Cord Contusion: A Comparative Study on Small and Large Animal Models

**DOI:** 10.3390/biom9120811

**Published:** 2019-12-01

**Authors:** Yana Mukhamedshina, Iliya Shulman, Sergei Ogurcov, Alexander Kostennikov, Elena Zakirova, Elvira Akhmetzyanova, Alexander Rogozhin, Galina Masgutova, Victoria James, Ruslan Masgutov, Igor Lavrov, Albert Rizvanov

**Affiliations:** 1 Clinical Research Center for Precision and Regenerative Medicine, Institute of Fundamental Medicine and Biology, Kazan Federal University, 420008 Kazan, Russia; ilyashul@mail.ru (I.S.); sergogurtsov@rambler.ru (S.O.); maldito.goldpride@gmail.com (A.K.); lenahamzina@yandex.ru (E.Z.); elyaelya18@gmail.com (E.A.); alex_rogozhin@mail.ru (A.R.); galina2526@gmail.com (G.M.); masgut@gmail.com (R.M.); igor.lavrov@gmail.com (I.L.); rizvanov@gmail.com (A.R.); 2 Department of Histology, Cytology, and Embryology, Kazan State Medical University, 420012 Kazan, Russia; 3 Republic Clinical Hospital, 420138 Kazan, Russia; 4 Department of Neurology, Kazan State Medical Academy–Branch Campus of the Federal State Budgetary Edicational Institution of Father Professional Education «Russian Medical Academy of Continuous Professional Education», 420012 Kazan, Russia; 5 Division of Biomedical Science, School of Veterinary Medicine and Science, Faculty of Medicine and Health Sciences, University of Nottingham Biodiscovery Institute, University Park, Nottingham NG7 2RD, UK; Victoria.James@nottingham.ac.uk; 6 Department of Neurologic Surgery, Mayo Clinic, Rochester, MN 55905, USA; 7 Department of Physiology and Biomedical Engineering, Mayo Clinic, Rochester, MN 55905, USA

**Keywords:** spinal cord injury, mesenchymal stem cells, adipose tissue, bone marrow, dental pulp, fibrin matrix, rat, pig

## Abstract

Here, we provide a first comparative study of the therapeutic potential of allogeneic mesenchymal stem cells derived from bone marrow (BM-MSCs), adipose tissue (AD-MSCs), and dental pulp (DP-MSCs) embedded in fibrin matrix, in small (rat) and large (pig) spinal cord injury (SCI) models during subacute period of spinal contusion. Results of behavioral, electrophysiological, and histological assessment as well as immunohistochemistry and real-time polymerase chain reaction analysis suggest that application of AD-MSCs combined with a fibrin matrix within the subacute period in rats (2 weeks after injury), provides significantly higher post-traumatic regeneration compared to a similar application of BM-MSCs or DP-MSCs**.** Within the rat model, use of AD-MSCs resulted in a marked change in: (1) restoration of locomotor activity and conduction along spinal axons; (2) reduction of post-traumatic cavitation and enhancing tissue retention; and (3) modulation of microglial and astroglial activation. The effect of an autologous application of AD-MSCs during the subacute period after spinal contusion was also confirmed in pigs (6 weeks after injury). Effects included: (1) partial restoration of the somatosensory spinal pathways; (2) reduction of post-traumatic cavitation and enhancing tissue retention; and (3) modulation of astroglial activation in dorsal root entry zone. However, pigs only partially replicated the findings observed in rats. Together, these results indicate application of AD-MSCs embedded in fibrin matrix at the site of SCI during the subacute period can facilitate regeneration of nervous tissue in rats and pigs. These results, for the first time, provide robust support for the use of AD-MSC to treat subacute SCI.

## 1. Introduction

Mesenchymal stem cells (MSCs) have to date been one of the most promising progenitor cell types, associated with diverse functional capabilities and extensive tissue regenerative potential. MSCs can migrate to areas of injury [1] and integrate with damaged tissues, where they mediate immunomodulatory [2,3,4], antiapoptotic, and anti-inflammatory effects through the secretion of various neurotrophic factors and cytokines and signaling pathway activation through specific receptors on target cells [5]. These specific features, along with multiple studies reporting the ability of MSCs to promote regeneration of nervous tissues in the brain and spinal cord, support the potential of MSCs as treatments of neurodegenerative conditions and traumatic brain and spinal cord injury (SCI) [6,7].

MSCs with specific immunophenotypic characteristics and differentiation potentials can be found in different organs. Bone marrow is the most common source of MSCs (BM-MSCs) used in studies of SCI at both preclinical and clinical stages [8]. There are several difficulties associated with the isolation of cells from the bone marrow, for example their number and regenerative potential, which decreases sharply with age. Another promising source of MSCs is adipose tissue (AD-MSCs), which due to the simple method of isolation and purification, alongside more consistent proliferative activity, gives AD-MSCs the potential to be more easily clinically translated. Furthermore, AD-MSCs were found to be effective in protecting the synaptic stability of motor neurons and in the facilitation of antioxidant and anti-inflammatory mechanisms after SCI [9,10]. Dental pulp-derived MSCs (DP-MSCs) have also been proposed as an alternative to BM-MSCs and AD-MSCs [11,12]. DP-MSCs were found to be effective in the protection of sensory and motor neurons after SCI, through the inhibition of apoptosis and promotion of axonal growth [13,14,15].

The main features of all MSCs, including their fibroblast-like morphology, growth characteristics in culture, ability to differentiate into osteogenic, adipogenic, and chondrogenic lineages, and broad profiles of gene expression make them valuable tools for neuroregenerative studies [16,17,18,19,20]. However, a comparative analysis of the regenerative potential of MSCs obtained from different sources indicates there are differences. BM-MSCs have greater chondrogenic and osteogenic potentials, with a similar colony efficiency to AD-MSCs, but lack the biological advantages of AD-MSCs when comparing proliferative capacity and immunomodulatory effects [21,22,23]. DP-MSCs, in turn, have a significantly greater cytoprotective effects compared to BM-MSCs [24]. We hypothesize that differences in the features of MSCs due to tissue origin and donor-related variations in regenerative potential determine the overall regenerative and protective effects seen when applied as a treatment for SCI.

Systemic, intraspinal, and intrathecal routes of administration of cell-based treatments have several drawbacks. These drawbacks are primarily associated with limited injection volumes, low stem cell viability in situ, and side effects. Biopolymer matrices are commonly used to maintain transplanted cells and optimize the microenvironment to increase cell survival and to control distribution of the bioactive molecules released by the implanted cells [25,26,27]. The combined application of MSCs with such a matrix could provide a safe and effective approach for cell transplantation into humans with SCI [7,26,28].

Preclinical studies of cell-based therapies commonly utilize small laboratory animals (rats, mice) as models, with reported structural and functional restoration of damaged tissue. However, translation of the results to clinical trials do not always produce the same effect in humans, often due to structural differences between humans and small animals, as well as variations in physiological characteristics, mechanisms of disease development, and the inability to perform autologous transplantation [29,30,31]. Preclinical studies performed in larger animal models (for example pigs and monkeys) may circumvent some of these issues, as these species share more anatomical and functional similarities with humans. The use of larger animal models may facilitate better translation to human clinical trials, minimizing economic risks.

A major gap in the knowledge required for the successful translation of preclinical studies is the absence of adequate comparisons between different animal models, particularly between commonly used small and large species within the same study. To address this critical gap, we compared the therapeutic potential of allogeneic BM-MSCs, AD-MSCs, and DP-MSCs embedded in fibrin matrix (FM) in both rat and pig models of spinal cord contusion. Our results indicate that in the rat model of the subacute period of spinal contusion, AD-MSCs have a better neuroregenerative potential compared to MSCs from other tissue origins, based on criteria scoring functional recovery, tissue retention, reduction of post-traumatic cavitation, modulation of microglial and astroglial activation, and neuroprotection. Application of AD-MSCs in the subacute period of SCI in pigs also showed significant differences based on behavioral evaluation, with some improvement in the restoration of the somatosensory pathways, tissue retention, reduction of post-traumatic cavitation, and predominant modulation of astroglial activation in dorsal root entry zone.

## 2. Materials and Methods

**Figure 1:**
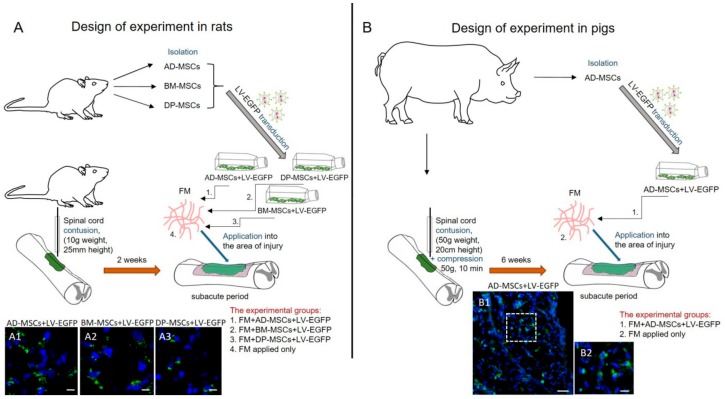
Study design in rodent and porcine models. (**A**) For experiments in rats, mesenchymal stem cells derived from bone marrow (BM-MSCs), adipose tissue (AD-MSCs), and dental pulp (DP-MSCs) were isolated, transduced by lentiviral vectors encoding enhanced green fluorescent protein (LV-EGFP) at passage 0 and cultivated up to passage 3. Two weeks after spinal cord injury (SCI) the obtained cells embedded in fibrin matrix (FM) were used for allogenic application into the area of injury. (**A1**–**A3**) Lower panels illustrate EGFP^+^-cells at day 14 after application of AD-MSCs, BM-MSCs, and DP-MSCs, transduced by LV-EGFP, in dorsal root entry zone (DREZ). Scale bar: 5 µm. (**B**) For experiments in pigs, AD-MSCs were isolated, transduced by LV-EGFP at passage 0 and cultivated up to passage 3. Six weeks after SCI the obtained cells embedded in FM were used for autologous application into the area of injury. (**B1**,**B2**) Lower panels illustrates EGFP^+^-cells at day 14 after application of AD-MSCs, transduced by LV-EGFP, in DREZ. The area marked in **B1** with dashed boxed areas; corresponds to the enlarged image B2. Scale bars: 50 (**B1**) and 10 (**B2**) µm.

The methods described herein were approved by the Kazan Federal University Animal Care and Use Committee (Permit Number 2, 5 May 2015) and experimental protocols were consistent with the guidelines of the Association for Assessment and Accreditation of Laboratory Animal Care International and Physiological Section of the Russian National Committee on Bioethics. All studies were designed to minimize animal numbers and the severity of procedures. Study designs are illustrated in Figure 1.

### 2.1. Isolation and Culture of Mesenchymal Stem Cells

For all cultured cells, the medium was replaced every 3 days. At passage 3, the cells were used for the subsequent experiments. MSCs were derived from the adipose tissue, bone marrow and dental pulp of female Wistar rats (weighing 250–300 g each; Pushchino Laboratory, Pushchino, Russia) and adipose tissue from 4-month-old female pot-bellied pigs (9–12 kg) followed isolation and characterization [32].

#### 2.1.1. Rat AD-MSCs 

Rat adipose tissue was collected aseptically in an operating room. Further manipulations were carried out in a cell culture laboratory as previously described [32]. The adipose tissue was minced, purified by centrifugation, and digested with a 0.2% crab hepatopancreas collagenase (Biolot, St. Petersburg, Russia) at 37 °C for 1 h with agitation. The homogenate was centrifuged and the enzymatic solution was decanted. The cell pellet was suspended with a Dulbecco’s phosphate-buffered saline (DPBS, PanEco, Moscow, Russia) solution and centrifuged to remove residual enzymes. The obtained cells were cultured in α-MEM medium with 10% fetal bovine serum (FBS), 100 U/mL penicillin, 100 μg/mL streptomycin, and 2 mM l-glutamine (all obtained from PanEco).

#### 2.1.2. Rat BM-MSCs 

BM-MSCs were collected from femurs. Briefly, normal regular Wistar rats were sacrificed, and their femurs were removed with surgical scissors. After removal of removal of residual soft tissue, epiphyses of the femurs were cut and the marrow was collected by flushing the canals with DPBS. The flushed solution was collected in 50 mL sterile tube and centrifuges 5 min at 500× *g*, after which the supernatant was removed. The cell pellet was resuspended in cultural medium, containing DMEM, 10% FBS, 100 U/mL penicillin, 100 μg/mL streptomycin, and 2 mM l-glutamine, and thoroughly homogenized. Cells were seeded into culture flasks and incubated for 24 h under standard conditions (37 °C and 5% CO_2_); the non-adherent cells were removed by replacing the culture medium.

#### 2.1.3. Rat DP-MSCs

DP-MSCs were harvested using a previously reported method [33]. Briefly, extracted teeth were removed and placed in sterile PBS containing 100 U/mL penicillin and 100 mg/mL streptomycin. The pulp was removed from the cavity, washed in sterile PBS, and minced with ophthalmology scissors. Centrifugation at 2000× *g* for 5 min, was used to pellet the cells. The supernatant was discarded and the cells were resuspended in 2.5 mL of culture medium (α-MEM, 10% FBS, 100 μM l-ascorbic acid 2-phosphate (Sigma-Aldrich, St. Louis, MI, USA), 2 mM l-glutamine, 100 U/mL penicillin, and 100 mg/mL streptomycin). Cells were seed into culture flasks and incubated at 37 °C and 5% CO_2_ for 3 days, avoiding any movement that may displace the cells.

#### 2.1.4. Pig MSCs 

Pig adipose tissue was collected aseptically from the subcutaneous fat of 4-month-old female pot-bellied pigs under anesthesia (propofol (2–6 mg/kg) via endotracheal intubation and maintained with isoflurane (1.3%)). Infiltration of the subcutaneous tissue was performed with a solution (total volume 200–300 mL) containing 1 mL of epinephrine hydrochloride (1 mg/mL) in 200 mL 0.9% NaCl. A small skin incision was made to a length of 0.5–1 cm, then the adipose tissue was cannulated by an endoscopic cannula (Eleps, Kazan, Russia, diameter 3 mm). The liquid and fat deposits were removed by vacuum extraction and subcutaneous fat collected in sterile 50 mL tubes containing 0.9% NaCl solution and delivered to the culture laboratory for subsequent cultivation.

The obtained subcutaneous fat was centrifuged at 500× *g* for 5 min, after which the supernatant was removed. The adipose tissue was thoroughly homogenized with sterile scissors and 0.9% NaCl solution was added. The centrifugation was repeated and the supernatant was removed. A freshly prepared sterile solution 0.2% crab hepatopancreas collagenase was added to the adipose tissue homogenate and incubated at 37 °C for 1 h with shaking. Then, the homogenate was centrifuged at 500× *g* for 5 min and the supernatant removed. Resulting cells (of stromal vascular fraction of adipose tissue) were seeded into culture flasks and cultivated in medium containing DMEM, 20% FBS, 2 mM l-glutamine, 100 μM l-ascorbic acid 2-phosphate, 100 U/mL penicillin, and 100 mg/mL streptomycin at 37 °C and 5% CO_2_. After 24–48 h, the non-adherent cells were removed by replacing the culture medium.

### 2.2. Lentiviral Transduction of MSCs

The MSCs at passage 0 were transduced with lentiviral vectors encoding enhanced green fluorescent protein (EGFP) (AD-MSCs + LV-EGFP, BM-MSCs + LV-EGFP, DP-MSCs + LV-EGFP) as previously described [7]. The percentages of EGFP-positive cells were assessed by flow cytometry (Guava EasyCyte 8HT, Millipore, MA, USA). After viral transduction, the MSCs, regardless or origin, typically begin to express EGFP within 48 h, with a plateau of expression reached at 96 h and 74 ± 5% of the MSCs in each population studied being EGFP-positive.

### 2.3. Flow Cytometry of MSCs

The cells were trypsinized and washed in PBS twice, pelleting cells after each wash by centrifugation at 500× *g* for 3 min. Cells were incubated with primary antibodies for 1 h at room temperature (RT). Subsequently, cells were washed three times with PBS, centrifuging at 500× *g* for 3 min to pellet the cells between washes, and incubated with secondary antibodiesfor a further 1 h at RT. After staining, the cells were fixed in 10% paraformaldehyde (PFA, Sigma-Aldrich) for 30 min and washed twice in PBS as detailed above. Cells were analyzed using a flow cytometer FACS Aria III (BD Biosciences, San Jose, CA, USA). The cells obtained from five animals were analyzed in each group, data are provided in Section 3.

### 2.4. Animals and Investigated Groups

Figure 1 provides a design of experiments in rats and pigs.

#### 2.4.1. Rats

One hundred fifteen adult female Wistar rats were randomly assigned to five groups. Allogenic MSCs + LV-EGFP, 1 × 10^6^ cells per rat, mixed with FM Tissucol (18 μL, Baxter, Deerfield, IL, USA) were applied on top of the injury through 2 weeks after SCI. The groups were: (1) FM + AD-MSCs + LV-EGFP, *n* = 25; (2) FM + BM-MSCs + LV-EGFP, *n* = 25; (3) FM + DP-MSCs + LV-EGFP, *n* = 25; (4) FM applied only, *n* = 25; and (5) without SCI (intact control, *n* = 15). All animals were evaluated by behavioral and electrophysiological assessment. Ten animals in each experimental group and seven in the intact control group were randomly selected for histology/immunohistochemistry and RT-PCR analysis. Animals were housed per group in clear plastic cages (12 h:12 h light/dark cycle) with food and water available ad libitum.

#### 2.4.2. Pigs 

Seventeen healthy 4-month-old female pot-bellied pigs (9–12 kg) were used in this study. All pigs were clinically judged to be in good health and to have a normal neurological status. Autologous AD-MSCs + LV-EGFP, 8 × 10^6^ cells per pig, mixed with FM Tissucol (150 μL) was applied on top of the injury through 6 weeks after SCI. The groups were: (1) FM + AD-MSCs + LV-EGFP, *n* = 5; (2) FM applied only, *n* = 5; and (3) without SCI (intact control, *n* = 5). Two additional pigs were used to evaluate the distribution of AD-MSCs in the area of SCI at day 14 after application.

### 2.5. Surgical Procedures

#### 2.5.1. Rats 

Rat SCI and cell application were carried out as previously described [7]. Rats were deeply anesthetized under general anesthesia with isoflurane and laminectomy at the Th8 vertebral level was performed. Zoletil (20 mg/kg, Virbac Sante Animale, France) was used by intramuscular injection for analgesia. Moderate spinal cord contusion was induced with an impact rod (2 mm diameter, 10 g) and a custom-made weight drop device (impactor) [7], which centered at the Th8 and dropped from 25 mm height. After SCI, the wound was sutured in layers. Following surgery, the rats had intramuscular injections of gentamicin (25 mg/kg, Microgen, Moscow, Russia) for 7 consecutive days. The injured rats’ bladders were manually emptied twice daily until spontaneous voiding occurred.

The skin was re-incised to expose the spinal cord 2 weeks after SCI (subacute period). After removing synechiae and making several longitudinal notches in the dura mater, FM with or without MSCs were applied on top of the injury and the dorsal back muscles and the skin were sutured. After surgery, the rats received daily intramuscular doses of gentamicin (25 mg/kg) for 7 consecutive days. After the operation, the rats were monitored and following evaluation showed consistent and similar changes in motor performance on both legs. For cell application, we strictly selected the spinal cord contusion model that showed complete hind limb paralysis.

#### 2.5.2. Pigs 

After intramuscular injection of xylazine (0.6 mg/kg) and ketamine (5 mg/kg), anesthesia was induced with propofol (2–6 mg/kg) and maintained with isoflurane (1.3%) during the operation. Laminectomy was performed at vertebral level Th10 and spinal cord contusion was induced by impact rod (7 mm diameter, 50 g) from 20 cm height, followed by 10 min of compression [34]. Due to the expected variation in spinal cords in pigs, X-ray assessment of thoracic and lumbar levels was performed to identify the same level for SCI across all animals. To approximate the degree of damage to a human SCI, sustained compression was carrying out (10 min) in addition to contusion to mimic prolonged spinal compression commonly found in traumatic SCI in human [34]. Following injury, the back muscles and the skin were sutured in layers. A urinary catheter (10 Fr, Jorgensen Laboratories Inc., Loveland, CO, USA) was manually inserted for postinjury bladder drainage and removed 3–5 days after SCI, after which the animals were able to reflexively empty their bladders. Animals were given cefazolin (25 mg/kg, Sintez, Kurgan, Russia) and ketoprophen (1 mg/kg, AVZ, Moscow, Russia) via intramuscular injection after surgery and for 5 days, and were fasted for at least 12 h after surgery. Pigs were housed individually for the first 48 h, after which they were housed in pairs.

Under general anesthesia, the skin was re-incised to expose the spinal cord 6 weeks after SCI in pigs (subacute period). The selection of the subacute period of cell transplantation was due to the technical possibility of carrying out autologous MSCs transplantation in a clinical practice, when there are surgical indications for reoperation, during which it is possible to apply MSCs obtained from a patient in an acute period and cultivate to the required amount. After removing synechias and making several longitudinal notches in the dura mater (2–3 notches, 1–2 mm length), FM with or without MSCs were applied on top of the injured spinal cord. The wound was sutured and pigs received intramuscular doses of cefazolin (25 mg/kg) and ketoprophen (1 mg/kg) for 5 days.

### 2.6. Behavioral Assessment

All behavioral studies were scored simultaneously by two observers who were blinded to the treatment groups. Final scores were obtained by averaging the two scores awarded by the examiners.

#### 2.6.1. Rats 

Motor function was evaluated using the open-field Basso, Beattie, Bresnahan (BBB) [35] locomotor rating scale. The baseline was obtained at 3 days before SCI. BBB rating scale defines three phases for the recovery of voluntary movements after spinal cord contusion: early (scores of 0–7), intermediate (scores of 8–13), and late (scores of 14–21). To evaluate differences in functional recovery, a behavioral assessment in the experimental groups was performed before SCI, on day 1, and once a week up to 11 weeks after SCI.

#### 2.6.2. Pigs 

For assessment of the motor function restoration in pigs, the Porcine Thoracic Injury Behavioral Scale (PTIBS) was used [34]. PTIBS is a 10-point scale, where the lowest scores (1–3) describe varying degrees of “dragging”, and the highest scores (7–10) describe walking behavior. Videotaping of locomotor recovery in the experimental groups was performed before SCI, on day 3, and once a week up to 22 weeks after SCI. All pigs were walking through a 50-m-long corridor within a 15 min time-period, while recorded on video camera.

### 2.7. Electrophysiological Studies

Electrophysiological tests were performed under anesthesia on intact rats and experimentally injured rats at 2 and 11 weeks after SCI as previously described [7,36]. Electrophysiological tests were performed under anesthesia on experimental pigs before the injury, at 15 min, and at 6 and 22 weeks after SCI. The M- and H-waves (pigs only have M-response) from the tibialis anterior muscle in pigs and the gastrocnemius muscle in rats were recorded in response to stimulation of the sciatic nerve [37,38].

Monopolar needle electrodes were used for recording. An active electrode was inserted into the middle of the muscle belly and the reference electrode was implanted within a region of the tendomuscular junction. Electrical stimulation of the sciatic nerve was performed with square-wave single stimuli and a pulse width of 0.2 ms. A skin electrode with fixed electrode spacing and monopolar needle electrodes inserted subcutaneously within an area where the sciatic nerve exits from the pelvis were used for stimulation in pigs and rats, respectively [39].

Transcranial electrical stimulation (TES) was carried out for registration of motor evoked potentials (MEPs) recorded by subcutaneous needle electrodes in tested muscles. The recording electrodes remained in the tibialis anterior muscle or the gastrocnemius muscle. TES was performed by needle electrodes inserted under the scalp up to the contact with the bone of the skull. The cathode was placed in the middle, approximately 0.5 cm caudally from the interorbital line, and the anode was placed in the middle near the occipital bone. Duration of stimuli ranged from 0.04 (rats) to 0.1 (pigs) ms, with an intensity from 20 to 500 V. An average of three repetitions and close in latency responses were analyzed and compared.

Somatosensory evoked potentials (SEPs) were registered by monopolar needle electrodes inserted subcutaneously for evaluation of conduction across the posterior columns of the spinal cord. An active electrode was inserted over upper lumbar vertebrae and a reference electrode was inserted over middle thoracic vertebrae for registration from lumbar level. The cathode electrode was also inserted in the middle, approximately 0.5 cm caudally from the interorbital line in rats and over the vertex in pigs, and the anode electrode in the middle near the occipital bone in rats and over the snout in pigs for registration from scalp. Electrical stimulation was performed using round electrodes in rats. Stimulation of the tibial nerve in the medial malleolus area was performed with a cutaneous stimulating electrode with a fixed interelectrode distance in pigs. Rectangular electric stimuli 0.2 ms with frequency of 3 Hz were used. The stimulus intensity was chosen based on the tail movements in rats and foot muscles in pigs (smallest stimulus producing tail movements was used). An average of three repetitions and close in latency responses were analyzed and compared.

### 2.8. Histology and Immunohistochemistry

At 7, 14, 30 and 60 days after reoperation, rats were anesthetized and subjected to intracardiac perfusion with 4% PFA (4 °C). Spinal cord pieces (50 mm segment centered around the injury site) were removed, fixed in 4% PFA at 4 °C overnight and incubated in 30% sucrose. At 2 and 16 weeks after reoperation, pigs were deeply anesthetized and transcardially perfused with 4% PFA (4 °C). An 8 cm segment of thoracic spinal cord centered around the injury site was collected, fixed in 4% PFA for 48 h, divided into 1-cm blocks in length and cryoprotected in 15% and 30% sucrose. Samples were embedded in tissue freezing medium (Tissue-Tek O.C.T. Compound, Sakura, Torrance, CA, USA) and then 20 μm transverse tissue sections were obtained using the cryostat Microm HM 560 (Thermo Scientific, Waltham, MA, USA).

A series of cross-sections obtained at day 60 in rats and week 16 in pigs after reoperation were used. Tissue sections prepared from spinal cord regions just 5 mm rostral and caudal to the injury epicenter were stained with Azur-eosin (MiniMed, Syponevo, Russia). Images were then captured with a light scanning microscope APERIOCS2 (Leica, Allendale, NJ, USA). The cross-sectional area of the spared tissue and abnormal cavities was measured. A total area of abnormal cavities in the spinal cord cross-section was calculated by counting cysts with an area of not less than 1.500 µm^2^. Aperio ImageScope software 12.4 (Leica) was used to measure the tissue area.

For immunofluorescence staining, sections were blocked with 5% normal goat serum for 1 h at RT and then incubated with primary antibodies overnight at 4 °C (Table 1). For visualization, fluorophore-conjugated secondary antibodies were applied for 2 h at RT. After washing, 4′,6-diamidino-2-phenylindole (DAPI) (10 μg/mL in PBS, Sigma) was used to visualize the nuclei. Coverslips were mounted on slides using mounting medium (ImmunoHistoMount, Santa Cruz, Dallas, TX, USA) and the stained sections were examined using an LSM 780 Confocal Microscope (Carl Zeiss, Jena, Germany). Using the Zen 2012 Software (Carl Zeiss) we analyzed the total intensity of GFAP and Iba1 (semiquantitative analysis) at 5-mm increments extending from the contusion center of the SCI. All sections were imaged using identical confocal settings (laser intensity, gain, and offset). The areas selected for a semiquantitative immunohistochemical evaluation as previously described were the ventral horns (VH), the dorsal corticospinal tract (CST, except the pigs), ventral funiculi (VF), the area around the central canal (CC), and the dorsal root entry zone (DREZ) ([40]). The analysis was not carried out in the CST area in pigs due to the presence of cavities in this site in control group after application of FM only.

### 2.9. Real-Time PCR

Total RNA was isolated from fresh rat spinal cords (5-mm-long segment encompassing the injury site) using a modified phenol/chloroform extraction and Yellow Solve Kit (Silex, Moscow, Russia) according to the manufacturer’s recommendations. Gene-specific primers and probes were designed and the selected sets of primers/probes were blasted against the GenBank to confirm their species and gene specificity. First strand cDNA synthesis was performed using 100 ng of total RNA, 100 units of RevertAid reverse transcriptase (Thermo Fisher Scientific), 100 pmol of random hexamer primers and 5 units of RNAse inhibitor according to the recommended manufacturer’s protocol. A quantitative analysis of mRNA of *18S, Gfap, Vimentin, S100, Pdgfα, Pdgfβ, Vegf, Fgf2, Hspa1b, CNPase, Ngf, Iba1, Mpz, Olig2, Caspase3, Mbp,* and *Gap-43* genes was performed using CFX 96 Real-Time PCR System (Bio-Rad, Hercules, CA, USA). We analyzed 100 ng of cDNA for expression of target genes, using 2.5× Reaction Mixture B (Syntol, Moscow, Russia), 200 nM of each primer and 100 nM probe (Supplementary Table S1). The mRNA expression was normalized using 18S rRNA. Plasmid DNA with corresponding inserts were used to perform standard curves. The mRNA level in intact spinal cord at Th8 level was considered as 100%. All RT-PCRs were performed in triplicate.

### 2.10. Cytokine Assay

Multiplex analyses based on the xMAP Luminex technology were performed using MILLIPLEX MAP Porcine Cytokine/Chemokine (magnetic) kit # PCYTMG-23K-13PX (Millipore), according to the manufacturer’s instructions. Experiments were performed in triplicate. The kit enables a simultaneous multiplex analysis of 13 pig cytokines/chemokines/interleukins (GM-CSF, IFN-γ, IL-1α, IL-1β, IL-1Rα, IL-2, IL-4, IL-6, IL-8, IL-10, IL-12, IL-18, TNF-α) in a 25-µL aliquot of porcine cell culture supernatant at passage 3.

### 2.11. Statistical Analysis

All statistics were calculated using Origin 7.0 SR0 Software (OriginLab, Northampton, MA, USA). Data are presented as mean ± standard deviation (SD). To determine statistical significance of the behavioral, electrophysiological, and morphometric data between experimental groups, which included different numbers of animals, we used nonparametric methods for testing whether samples originate from the same distribution. A one-way analysis of variance (ANOVA) with Tukey’s test or two-way analysis of variance (ANOVA) were used in this study for multiple comparisons between tested groups. All analyses were performed in a blinded manner with respect to the treatment group. A value of *p* < 0.05 was considered statistically significant.

## 3. Results

### 3.1. Characterization of Rat Mesenchymal Stem Cells

Analyses of the expression of surface markers in primary cultures of AD-MSCs, BM-MSCs, and DPMSCs, native and transduced with LV-EGFP, by flow cytometry were performed (Table 2). The expression of CD34 and CD45 were below the threshold <2% in all obtained cell cultures. The expression of CD90 and CD73, considered to be predominant MSC markers, were highest in AD-MSCs 99.9 ± 0.17%/98.6 ± 2.3% and 94 ± 0.5%/87.5 ± 4.4%, respectively. The level of CD90 expression was greater than 90% for all tested cultures, however, a significantly lower (*p* < 0.05) value was found in native and transduced by LV-EGFP DPMSCs compared to AD-MSCs and BM-MSCs. BM-MSCs and DP-MSCs cultures had reduced expression of CD29 (*p* < 0.05) compared to AD-MSCs. CD44 expression was significantly reduced (*p* < 0.05) in cultures of both native and transduced LV-EGFP DP-MSCs compared to BM-MSCs and AD-MSCs. It should be noted that transduction by LV-EGFP AD-MSCs, BM-MSCs, and DP-MSCs did not lead to significant changes in the expression of the tested markers.

### 3.2. Distribution and Survival of MSCs in the Area of SCI in Rats

A comparative analysis of the migration and viability of AD-MSCs, BM-MSCs, and DP-MSCs transplanted into the area of SCI was conducted. In the spinal cord of rats, transplantation of transduced by LV-EGFP AD-MSCs, BM-MSCs, and DP-MSCs produced specific fluorescence up to 5 mm rostrally and caudally from the injury site up to 60 days after cells were applied.

**Figure 2:**
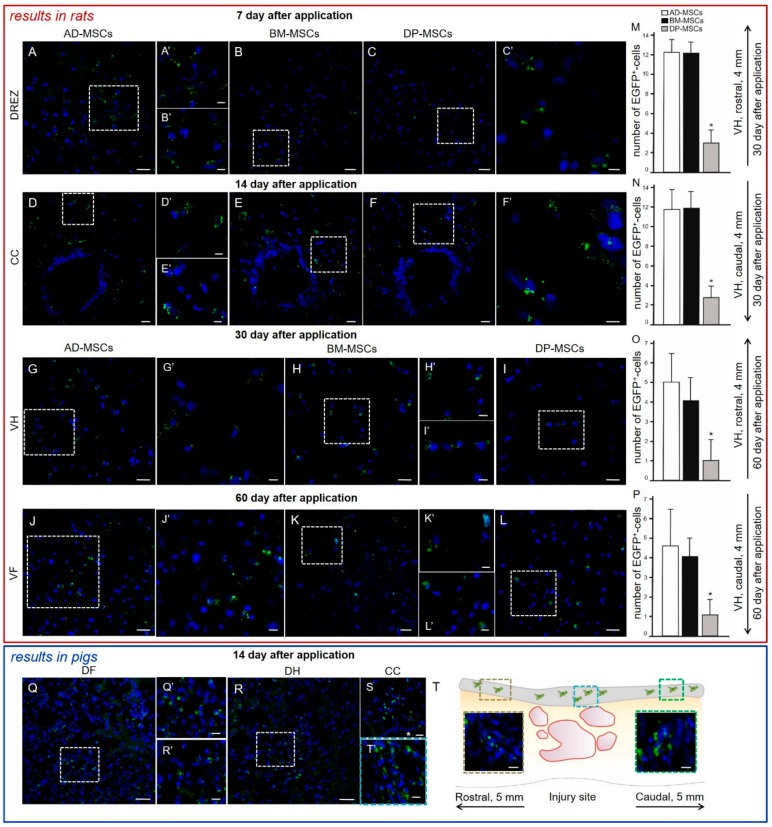
Distribution and survival of MSCs encapsulated in FM after application into the area of SCI in rats (red box) and pigs (blue box). MSCs predominantly migrate through the posterior roots of the spinal cord. In the early periods (7–14 days post-transplantation) labeled by LV-EGFP (green) AD-MSCs, BM-MSCs, and DP-MSCs were located mainly in (**A**–**C**) DREZ and (**D**–**F**) central canal (CC) in rats. During late periods (30–60 days post-transplantation) labeled LV-EGFP cells were also observed in the (**G**–**I**) ventral horns (VH) and (**J**–**L**) ventral funiculi (VF) in rats. The (**A**–**L**) areas marked with a dashed boxed correspond to enlarged images **A’**–**L’**. Number of EGFP^+^-cells at days (**M**,**N**) 30 and (**O**,**P**) 60 at 4 mm rostrally and caudally from the injury site in VH of experimental groups with MSCs transplantation. At day 14, LV-EGFP MSCs (green) were mainly located in the area of DREZ, (**Q**) dorsal funiculi (DF), (**R**) dorsal horns (DH), and (**S**) CC in pigs. The **Q** and **R** areas marked with a dashed boxes correspond to enlarged images **Q’** and **R’**, respectively. (**T**) MSCs distribution can be seen in the area of epidural fibrosis (gray line above spinal cord longitudinal section) according to the distance from injury site. MSCs were also detected in the area of epidural fibrosis, where more MSCs were found above the epicenter of injury and in the caudal direction, and less in the synechias above the rostral part of spinal cord. Asterisk indicates CC. Color dashed boxes (brown—rostral part; blue—epicenter of injury; green—caudal part) in scheme indicate corresponding area with MSCs distribution. Nuclei are stained with DAPI (blue). Scale bars: (**A**–**L**) 20, (**H’**) 10, and (**A’**–**G’,I’**–**J’**–**L’**) 5 μm; (**Q**,**R**) 50, (**S**) 20 and (**Q’**,**R’**,**T’**,**E**’, brown and green dashed boxes) 10 μm.

At days 7 and 14, labeled EGFP AD-MSCs, BM-MSCs, and DP-MSCs were predominantly located in DREZ, CC, and lateral CST (Figure 2A–F) and single fluorescent cells were present in the dorsal CST. There was no significant difference in the number and distribution of fluorescent MSCs between the experimental groups at these time points. At days 30 and 60, labeled EGFP cells were also found in the VH and VF (Figure 2G–L). Treated animals showed tropism of transplanted MSCs to the gray matter and their preferential migration through dorsal roots of the spinal cord; this can be attributed to directional paths for their movement and the effect of the SCI as disruptive barrier in this area [32]. The distribution of MSCs in the SCI area was similar for all three experimental groups.

No significant differences were found between the experimental groups in regard to the number of fluorescent cells in the white and gray matter in the early periods after SCI. At days 30 and 60, both rostrally and caudally from the injury, the FM + DP-MSCs group showed a decrease in the number of EGFP-labeled cells of ~4.1-fold (*p* < 0.05) in VH (Figure 2M–P) and their absence in CC compared to groups with AD-MSCs and BM-MSCs was detected.

### 3.3. Behavioral Results in Rats

**Figure 3:**
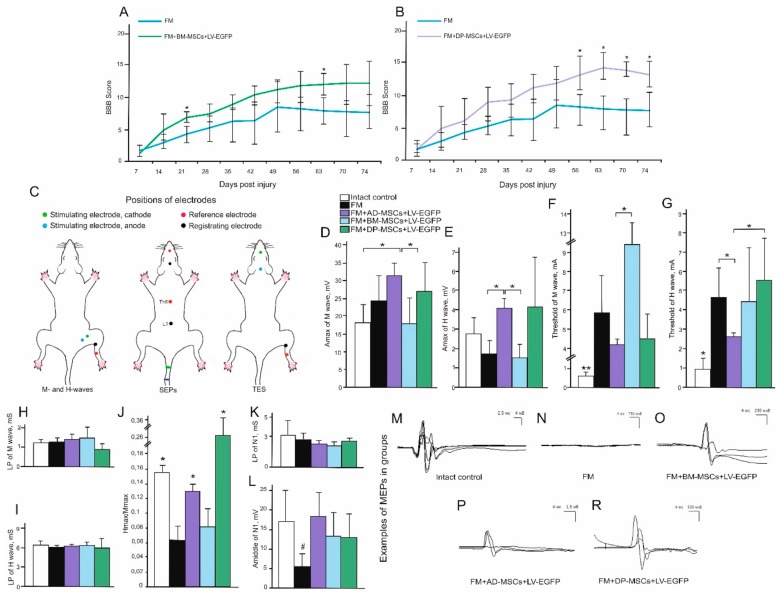
Behavioral and electrophysiological studies after SCI in experimental groups of rats. BBB locomotor scores of rats of the FM only (blue line), FM + BM-MSCs + LV-EGFP (green line), and FM + DP-MSCs + LV-EGFP (purple line) groups (**A,B**). The motor function scores were significantly higher after AD-MSCs and DP-MSCs application than those in the FM group 8 and 4 weeks postinjury, respectively. The lowest results in locomotor recovery between groups with MSC therapy were shown after application of BM-MSCs, where average BBB scores were higher at 3 and 9 weeks postinjury and not significantly different on week 11 compared to control group. * *p* < 0.05 as compared with the control group (FM), one-way ANOVA followed by a Tukey’s post hoc test. Schematic illustration of electrodes positions in rats (**C**). Amax of M- (**D**) and H-wave (**E**), threshold of M- (**F**) and H-waves (**G**), LP of M- (**H**) and H-waves (**I**), H/M wave amplitude ratio (**J**), LP (**K**) and Amiddle (**L**) of lumbar N1 after SCI in the experimental groups (X axis). At day 74 after SCI, the positive dynamics of the ratio Hmax/Mmax responses restoration was marked in groups with AD-MSCs and DP-MSCs application. At the same time, there was a decrease in Amax of N1 in the FM only group. * *p* < 0.05; ** *p* < 0.01; # *p* < 0.05 as compared with intact control and experimental group treated with AD-MSCs (one-way ANOVA followed by a Tukey’s post hoc test). Electrophysiology results demonstrate motor evoked potentials (MEPs) before SCI (**M**) and on week 11 in FM (**N**), FM+BM-MSCs+LV-EGFP (**O**), FM+AD-MSCs+LV-EGFP (**P**), and FM+DP-MSCs+LV-EGFP (**R**) groups.

A comparative assessment of locomotor activity after application of AD-MSCs, BM-MSCs, and DP-MSCs was performed using the open-field BBB locomotor rating scale. Previously, we observed on week 11 after SCI, the motor function scores were higher in groups with AD-MSCs (17.1 ± 3.1) compared to the control (FM only) group [7]. Significant differences (*p* < 0.05) between AD-MSCs and control FM group were also seen during weeks 4–11 postinjury and between AD-MSCs and BM-MSCs at 4–6 weeks postinjury (Figure 3A). During the last 4 weeks of the study, an increase (*p* < 0.05) of the BBB scores in groups treated with DP-MSCs compared to control FM group (Figure 3B) was detected. The lowest results on locomotor recovery among the experimental groups were found after treatment with BM-MSCs, where the average BBB score in rats was higher (*p* < 0.05) at 3 and 9 weeks postinjury and did not change significantly on week 11 compared to the control group with FM application only (Figure 3A). These findings suggest recovery of motor function following treatment with FM-embedded MSCs obtained from all three sources.

### 3.4. Electrophysiological Findings

#### Rats

M- and H-responses were recorded and the following parameters were evaluated across experimental groups: threshold, latency period (LP), maximum amplitude of the responses (A_max_), and the ratio of H-wave A_max_ and M-wave A_max_ (H_max_/M_max_) (Figure 3C–J). At day 74, in the group treated with AD-MSCs, a statistically significant increase of A_max_ (*p* < 0.05) compared to intact control and FM + BM-MSCs treated animals was found (Figure 3D). Previously, we hypothesized that the application of AD-MSCs would increase the number of muscle fibers. However, the morphometry of the calf muscles did not reveal any significant changes in the number of muscle fibers between the control and experimental groups [7].

H-response was absent in intact rats in 33% of cases [41] and in groups treated with AD-MSCs, BM-MSCs, and DP-MSCs H-response was absent in 40, 37.5, and 25% of cases, respectively. At the same time, in the control group with FM application only, the H-response was recorded in all cases, indicating facilitation of monosynaptic circuit following injury, which could be partially inhibited by supralisional influences in control and treated animals. At day 74 after SCI, the LP of H-wave remained unchanged in all investigated groups and a positive trend in the recovery (*p* < 0.05) of the H_max_/M_max_ response rate was found in groups with AD-MSCs and DP-MSCs (Figure 3J), which could reflect the recovery from spinal shock.

In intact rats, MEPs were recorded with an average amplitude 17.66 ± 5.30 mV and latency 5.81 ± 0.63 ms (Fig.3M). In the experimental groups, most of the animals showed bilateral MEPs, although in two rats MEPs were found only on one side. The average amplitude and latency values registered by MEPs in the experimental groups are presented in Table 3. In general, in groups treated with MSCs, a better recovery of the electrophysiological parameters was observed compared to the control group treated with FM only (Figure 3N–R). Comparing the ratio of the amplitude of the M-response to MEPs, all experimental groups with SCI were significantly different from the intact control, which is a sign of incomplete recovery. At the same time, the best indicators of MEPs recovery were noted in the groups with application AD-MSCs and BM-MSCs compared to other groups with SCI.

SEPs in intact rats from the lumbar level were presented by P1–N1–P2 complex, with the largest in amplitude peak N1. In intact control, average value of A_middle_ for peak N1 was 16.72 ± 8.68 µV; the average value for LP was 3.08 ± 0.97 ms (Figure 3K,L). N1–P1–N2–P2 complex (where N2 and P2 could be not registered in normal rats) was recorded with electrodes located on the scalp and A_middle_ and LP values of the N1 and P1 peaks were measured as well. An average value of A_middle_ for the peak of N1 in intact control was 3.11 ± 0.60 µV and the average value for LP was 7.85 ± 0.61 ms. An average value of A_middle_ for the peak of P1 in intact control was 4.65 ± 1.87 µV and the average value for LP was 14.65 ± 1.27 ms. The complex, recorded from the lumbar level, remained intact in all groups with SCI. However, there was a significant decrease of the A_middle_ of N1 (*p* < 0.05) in animals treated with FM only compared to intact control and group treated with AD-MSCs. The expected origin of peaks recorded from the lumbar region are the spinal roots and neurons of the posterior horns of the lumbar and sacral segments of the spinal cord. SCI at the thoracic level could affect these structures via edema and secondary circulatory changes, which could lead to a decrease in the A_middle_ of the lumbar peak. The absence of these changes in the group treated with AD-MSCs suggests the positive effect of AD-MSCs. In contrast to the TES results, there was practically no recovery of scalp SEPs. We were able to register scalp SEPs only in one rat in the group with application AD-MSCs.

### 3.5. Spinal Cord Morphometry in Rats

**Figure 4:**
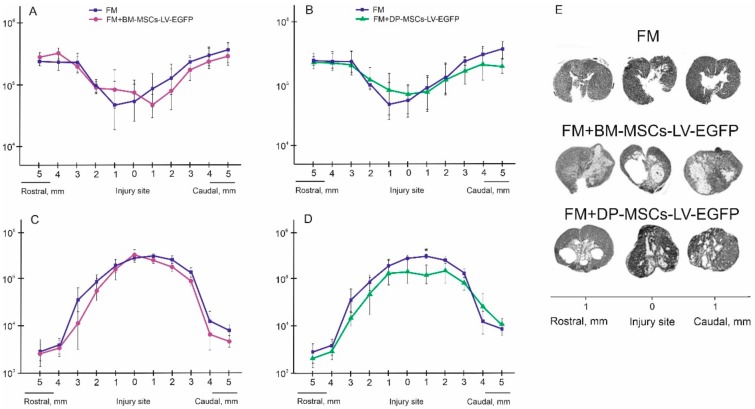
Spinal cord morphometry in experimental groups of rats. (**A**,**B**) An area of the spared tissue and (**C**,**D**) a total area of abnormal cavities, 5 mm rostrally and caudally from the injury epicenter at 60 day after treatment with FM (blue line), FM + BM-MSCs + LV-EGFP (purple line) or FM + DP-MSCs + LV-EGFP (green line). * *p* < 0.05, one-way ANOVA followed by a Tukey’s post hoc test. (**E**) Cross-sections of the injured spinal cord at 74 day after SCI in experimental groups at a distance of 1 mm rostrally and caudally from the injury epicenter. Azur-eosin staining. Previously obtained results in FM + AD-MSCs + LV-EGFP group are available in our article [7]

Previously, we observed that application of AD-MSCs leads to improvement of morphometric characteristics at day 60 after cell therapy, compared to the control (FM only) group: the area of spared tissue was larger at distances of 1 and 2 mm rostrally and 3 mm caudally from the injury epicenter (*p* < 0.05); the total area of abnormal cavities was less at a 1 mm distance rostrally and 1–3 mm caudally from the injury epicenter (*p* < 0.05) [7]. At the same time, experimental groups treated with BM-MSCs had no significant difference from the control (FM only) group within 5 mm in rostral and caudal directions (Figure 4A,C,E). After application DP-MSCs, the total area of abnormal cavities was significantly less at a distance of 1 mm caudally from the injury epicenter compared to the control (FM only) group (Figure 4B,D,E). A significant difference in the spared tissue was found between the groups treated with MSCs within an area 2 mm rostrally and 3 mm caudally from the injury epicenter. Based on earlier results and new data, we concluded that difference was greater in the FM + AD-MSCs group compared to FM + BM-MSCs. At 2 mm rostrally and 3.5 mm caudally from the injury epicenter, this difference was again greater in the FM + AD-MSCs group compared to FM + DP-MSCs. The total area of abnormal cavities in the group treated with AD-MSCs was smaller (*p* < 0.05) compared to BM-MSCs treated animals within the epicenter of injury and at a distance of 3 mm caudally from it; there was no significant difference with DP-MSCs group at all tested spinal cord regions.

### 3.6. Assessment of Astroglial and Microglial Cells in the Area of SCI in Rats

**Figure 5:**
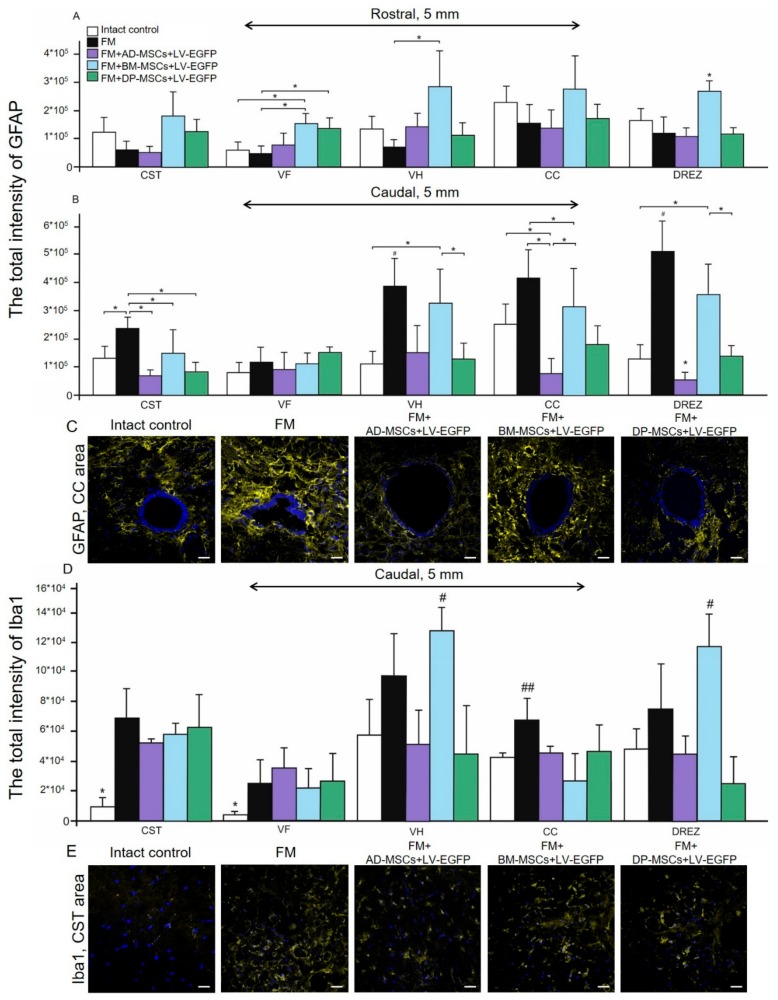
Assessment of astroglial and microglial activation at the site of injury in rats. The total intensity of (**A**,**B**) GFAP and (**D**) Iba1 (Y-axis) in the examined regions of the intact spinal cord (white column) or 60 day after treatment with FM only (black column), FM + AD-MSCs + LV-EGFP (violet column), FM + BM-MSCs + LV-EGFP (blue column), and FM + DP-MSCs + LV-EGFP (green column). * *p* < 0.05; # *p* < 0.05; and ## *p* < 0.05 as compared with all investigated groups, except FM and DP-MSCs; one-way ANOVA followed by a Tukey’s post hoc test. (**C**,**E**) Visualization of astroglial and microglial activation using GFAP (yellow) and Iba1 (yellow) 5 mm caudally from the injury epicenter within the CC and CST in the investigated groups. Nuclei are DAPI-stained (blue). Scale bar: 25 µm.

Using pan markers GFAP and Iba1 for astrocytes and microglia we produced a semiquantitative estimation of the cells in the areas of gray and white matter in spinal cord at a distance of 5 mm rostrally and caudally from the injury epicenter in all experimental groups (Figure 5). Astroglial activation was prominent (*p* < 0.05) in groups treated with BM-MSCs at a distance of 5 mm rostrally from the injury epicenter in comparison with other experimental groups in VH, CC, and DREZ (Figure 5A). At the same time, in the rostral direction we did not find significant differences in total intensity of GFAP between FM + AD-MSCs, FM + DP-MSCs, and FM treated groups, except VF for FM + DP-MSCs and FM groups (*p* < 0.05). In the caudal direction from the injury epicenter, differences between experimental groups in the level of GFAP expression were more prominent (Figure 5B). Significant changes between groups were observed in all investigated areas except VF. However, astroglial activation was more pronounced in the control (FM only) group and less pronounced in the group treated with FM + BM-MSCs. The total intensity of GFAP was least in the group treated with AD-MSCs, where in the regions of CC and DREZ this value was less (*p* < 0.05) compared to intact controls (Figure 5B,C).

Microglial activation was markedly upregulated after SCI primary at a distance of 5 mm caudally from the injury epicenter (Figure 5D). Peak values of the total intensity of Iba1 in grey matter VH and DREZ were detected in groups treated with FM + BM-MSCs or FM only. In white matter areas CST and VF, the total intensity of Iba1 was higher (*p* < 0.05) in all experimental groups compared to intact control (Figure 5D,E).

### 3.7. Analysis of mRNAs Expression in the Area of SCI in Rats

**Figure 6:**
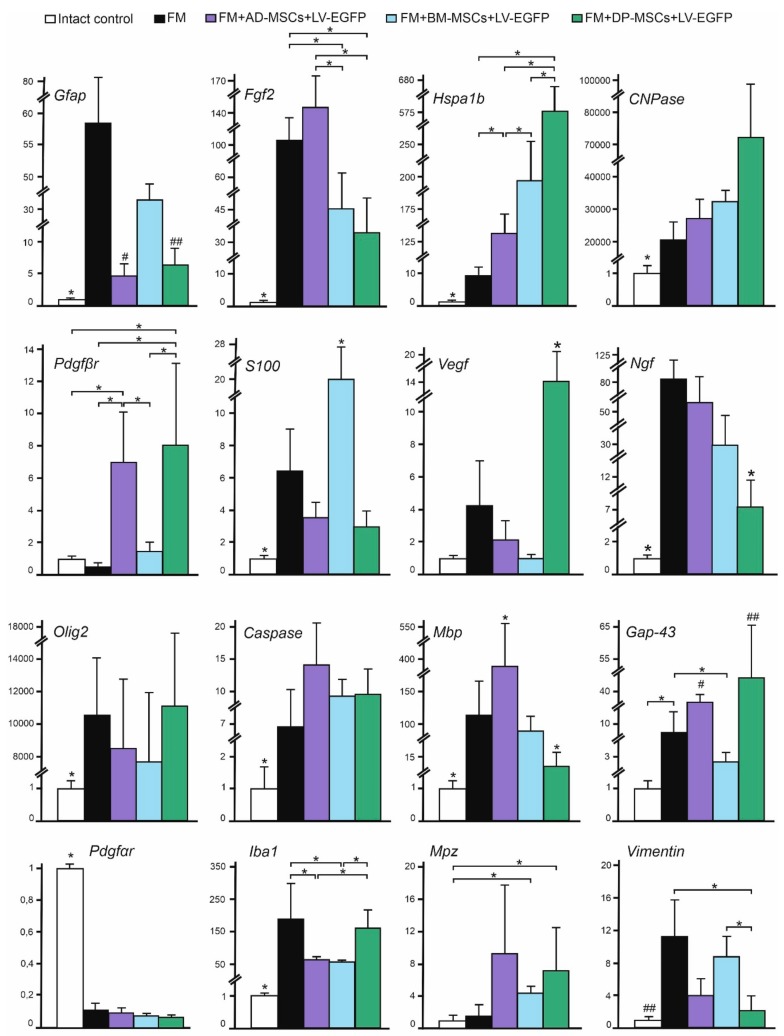
Analysis of mRNA expression in the area of SCI in rats. *Gfap, Fgf2, Hspa1b, CNPase, Pdgfβr, S100, Vegf, Ngf, Olig2, Caspase 3, Mbp, Gap-43, Pdgfαr, Iba1, Mpz,* and *Vimentin* mRNA expression in intact spinal cord (white column) and 60 days after FM (black column), FM + AD-MSCs + LV-EGFP (purple column), FM + BM-MSCs + LV-EGFP (blue column), or FM + DP-MSCs + LV-EGFP (green column) application. The mRNA expression levels in the intact spinal cord were considered as 100%. * *p* < 0.05; # *p* < 0.05 as compared with all investigated groups, except DP-MSCs; ## *p* < 0.05 as compared with all investigated groups, except AD-MSCs, one-way ANOVA followed by a Tukey’s post hoc test.

We performed qRT-PCR on sections obtained from the injury site at 74 day after SCI for all experimental groups (Figure 6). After SCI, increased expression of neurotrophic factor mRNA such as *Fgf2, Vegf,* and *Ngf* was observed in all experimental groups. Application of AD-MSCs led to the largest increase in mRNA expression of *Fgf2* and application of DP-MSCs lead to the largest increase in *Vegf* (*p* < 0.05), compared to other experimental groups. Variations in expression of neural cell marker mRNAs in injured spinal cords were found. These results demonstrate that the *Gfap* and *Iba1* mRNA expression was similar to those described previously using immunohistochemical analysis. We also observed increasing mRNA expression of myelin-related proteins such as *Mbp* and *Mpz;* the highest levels were found in AD-MSCs treated animals. The mRNA expression of *Pdgfβr* and *Gap-43* were markedly upregulated (*p* < 0.05) after AD-MSCs and DP-MSCs application when compared to other experimental groups and the intact control. Our results also demonstrate that the mRNA expression of vital antiapoptotic regulator *Hspa1b* was markedly upregulated in groups treated with MSCs, with the highest expression within the group treated with FM + DP-MSCs (*p* < 0.05).

### 3.8. Summary of Results from Rodent Model

Results described above suggest that application of AD-MSCs combined with FM in the subacute period stimulates post-traumatic regeneration to a greater extent compared to therapy with BM-MSCs and DP-MSCs. This conclusion is confirmed by: (1) recovery of locomotor activity and nerve fiber conduction obtained by electrophysiological data; (2) tissue sparing and reduction of post-traumatic cavitation; and (3) modulation of microglial and astroglial activation. Next, we tested the effect of AD-MSCs therapy in subacute period of SCI in large animals (pigs). Approximations for the degree of injury and experimental conditions were estimated to represent those used in rodents as closely as possible to allow for comparative assessment.

### 3.9. Characterization of Pig Mesenchymal Stem Cells Obtained from Adipose Tissue

The adherent cells in the primary culture were located singularly or in small groups at days 1–2 after seeding. On the 2 day of cultivation, the cells began to divide, forming islets. All cells were similar in morphology, being small in size and having an elongated (fibroblast-like) shape, in most cases with several processes. With further cultivation, the cells spread over the bottom of a culture flask and increased in size, whilst preserving the fibroblast-like morphology, a typical MSC morphology. As the number of cells in a culture increased, their processes became more closely adjoined to each other and the borders of individual cells were difficult to distinguish. Cultured cells were maintained for at least six passages.

Based on flow cytometry (before and after transduction), the expression of the following markers was determined: Thy-1 (CD90), 94.5 ± 3.7%/93.6 ± 5.2%; CD73, 92 ± 2.5%/91 ± 0.5%; CD44, 86.7 ± 8.9%/78 ± 4%; and CD29, 63 ± 9.5%/52.6 ± 13.5%. The expression of surface antigens CD34 and CD45 were not detected in AD-MSCs.

### 3.10. MSCs Cytokine Profile in Pig

Prior to autologous transplantation, AD-MSCs were labeled with GFP. In order to determine any changes in the secretory phenotype of AD-MSCs as a result of being transduced to express LV-EGP, we performed simultaneous multiple cytokine and chemokine analysis using xMap Luminex Bead-Based Multiplex Assays in the supernatant from porcine AD-MSC cultures before and after transduction with LV-EGFP (Table 4). We did not observe significant changes in the expression of the investigated markers in AD-MSCs after transduction by LV-EGFP.

### 3.11. Distribution and Survival of MSCs Transplanted into the Area of SCI in Pigs

We analyzed the behavior of MSCs at 14 days after application into the site of SCI in pigs and obtained results similar to those determined in the rat model. At this time, the cells were predominantly located in the area of DREZ, dorsal horns (DH), dorsal funiculi (DF), and CC (Figure 2Q–S). We noted that the synechias (epidural fibrosis) in pigs is more pronounced than in rats. In this regard, we also detected EGFP-labeled AD-MSCs in the area of epidural fibrosis, where more MSCs were found above the epicenter of injury and in the caudal direction, and less in the synechias above the rostral part of spinal cord (Figure 2T).

### 3.12. Behavioral and Electrophysiology Results in Pigs

**Figure 7:**
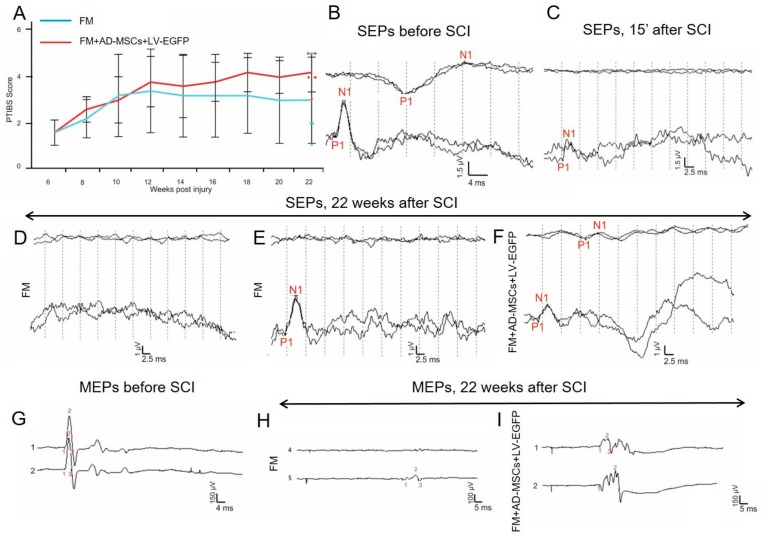
Behavioral and electrophysiology results in pigs. (**A**) PTIBS locomotor scores of pigs of the FM (blue line) and FM + AD-MSCs + LV-EGFP (red line) groups. Significant differences between the experimental groups throughout the experiment were not found. Electrophysiology results demonstrate SEPs (**B**) before SCI (reproducible peaks from lumbar and scalp electrodes) and (**C**) after 15 min after SCI (reproducible peaks of lesser amplitude from lumbar electrodes only). (**D**,**E**) At 22 weeks, in the control group with FM application, only one pig had a peak from lumbar enlargement on one side. (**F**) While in the experimental group with FM + AD-MSCs application, cortical peaks on one side and peaks from lumbar enlargement on both sides were recorded in one pig. MEPs from the tibialis anterior muscle in pigs (**G**) before SCI and after 22 weeks in groups with (**H**) FM and (**I**) AD-MSCs application with registration of MEPs on one side.

During the first week after SCI in pigs, no active hindlimb movements were observed, which corresponds to a score of 1 using the PTIBS scale (Figure 7A). At 6 weeks, before the application of FM only or FM + AD-MSCs, the animals had a score of 1.6 ± 0.5 (Supplementary Video S1). Over the next 16 weeks after reoperation, an increase of PTIBS scores, with the largest values in the group treated with AD-MSCs, was found. However, these differences were not statistically significant and at 22 weeks after SCI, the PTIBS scores in the FM only and FM + AD-MSCs groups were 4.2 ± 0.8 and 3 ± 1.8, respectively.

Electrophysiological data (M-response, MEPs, and SEPs) were recorded before the injury, at 15 min and at 6 and 22 weeks after SCI. No significant differences in the amplitude and latency of the M-response before and after the injury were found suggesting that the SCI at the thoracic level is not affecting motor neurons and motor axons of the lumbar segments. All animals before and after SCI (15 min) were evaluated for MEPs and SEPs (Figure 7B,C,G). At 6 weeks after SCI, no MEPs and scalp SEPs were recorded, with the exception of a single animal for which SEPs could be recorded from lumbar electrodes. These changes showed the absence of conduction along the lateral and posterior columns of the spinal cord, which indicated the adequacy of the injury model [42]. The absence of SEPs from the lumbar electrode in the majority of animals may indicate the spread of the SCI in the caudal direction.

Across all experimental groups, no difference in restoration of conductivity along the lateral columns was found; in the control group treated with FM only after 22 weeks only one pig had MEPs on one side (Figure 7H); and in the experimental group treated with FM + AD-MSCs during the same period MEPs were recorded in two pigs on one side (Figure 7I).

The main differences found relate to the ascending pathway of deep sensitivity of the spinal cord. At 22 weeks, in the control group with FM-only application, scalp SEPs were not found, and only one pig had a peak from lumbar enlargement on one side (Figure 7D,E). While in the experimental group treated with FM + AD-MSCs, cortical peaks on one side and peaks from lumbar enlargement on both sides were recorded in one pig (Figure 7F) and lumbar peaks were recorded on one side in two pigs. Thus, we can state the partial restoration of the somatosensory ways in three out of five pigs treated with FM + AD-MSCs.

### 3.13. Spinal Cord Morphometry in Pigs

**Figure 8:**
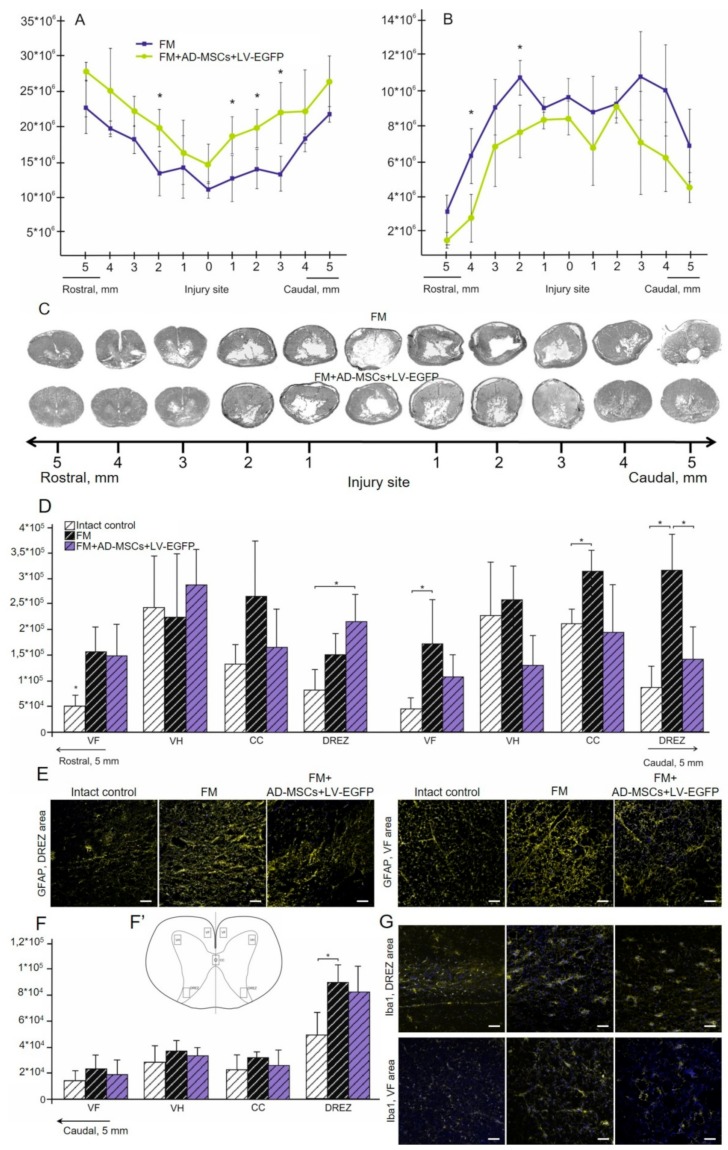
Spinal cord morphometry and assessment of astroglial/microglial activation at the site of injury in pigs. (**A**) An area of the spared tissue and (**B**) a total area of abnormal cavities, 5 mm rostrally and caudally from the injury epicenter in 16 weeks after FM (blue line) and FM + AD-MSCs + LV-EGFP (yellow line) application. * *p* < 0.05, one-way ANOVA followed by a Tukey’s post hoc test. (**C**) Cross-sections of the injured spinal cord 22 weeks after SCI in experimental groups at the distances of 5 mm rostrally and caudally from the injury epicenter. Azur-eosin staining. The total intensity of (**D**) GFAP and (**F**) Iba1 (Y-axis) in intact spinal cord (white column) or 16 weeks after FM (black column) and FM + AD-MSCs + LV-EGFP (violet column) application in the examined regions. * *p* < 0.05 as compared with all investigated or indicated groups, one-way ANOVA followed by a Tukey’s post hoc test. (**F’**) Schematic representation of the investigated areas. (**E**,**G**) Visualization of astroglial and microglial activation using GFAP (yellow) and Iba1 (yellow) 5 mm caudally from the injury epicenter within the DREZ and VF in the investigated groups. Nuclei are DAPI-stained (blue). Scale bar: 50 µm.

We observed significant difference in the areas of the spared tissue and abnormal cavities between the experimental groups (Figure 8). AD-MSCs application led to improvement of morphometric characteristics at 16 weeks after therapy compared to the control (FM only) group. The area of spared tissue was higher at a distance of 2 mm rostrally and 1–3 mm caudally from the injury epicenter (*p* < 0.05) and the total area of abnormal cavities was less at a distances of 2 and 4 mm rostrally from the injury epicenter (*p* < 0.05) (Figure 8A–C).

### 3.14. Assessment of Astroglial and Microglial Cells in the Area of SCI in Pigs

At 22 weeks after SCI, the level of GFAP expression in the FM only and FM + AD-MSC treated groups increased in comparison with intact control (Figure 8D). We observed significant difference between the control FM group and the FM + AD-MSCs group in DREZ caudally from the injury epicenter, where value of the total intensity of GFAP was more than 2-fold higher (*p* < 0.05) in FM control group (Figure 8D,E). After FM application, there were higher levels (*p* < 0.05) of GFAP expression relative to the intact controls in the VF and CC caudally from the injury epicenter, while the FM + AD-MSCs group showed no significant differences in analogical areas when compared with intact control or FM only treated pigs (Figure 8D,E).

Microglial activation was upregulated after SCI in experimental groups in DREZ at a distance of 5 mm caudally from the injury epicenter; a significant difference (*p* < 0.05) was identified between the FM-only group and intact control (Figure 8F,G). Iba1^+^-microglia in DREZ were mainly visualized as cell clusters in groups with SCI compared to intact control, in which microglia were distributed more evenly.

## 4. Discussion

Experimental approaches for the treatment of SCI have been evolving and currently include a variety of approaches with a significant prevalence of genetic [43,44,45] and cellular therapies. Cells of adult organism, embryonic cells, and induced pluripotent stem cells have been used in multiple studies with variable effects [46,47,48], as has the application of extracellular vesicles as a cell-free therapy [49]. In the context of safety and practical capacity of translation into the clinic, MSCs appear to be the most promising therapy at the current time [50]. In addition, FM application is a widespread neurosurgical technique commonly used in humans. In this study, the comparative efficiency of MSCs derived from bone marrow, adipose tissue, and dental pulp combined with FM in the subacute period of SCI was evaluated for the first time comparatively across small (rat) and large (pig) animal models.

Prior to in vivo experiments, we performed a morphological and phenotypic assessment of our cultivated MSCs. Results showed reduced CD29 expression in BM-MSCs and DP-MSCs cultures, as well as a significant reduction of CD44 expression in DP-MSCs, in comparison with AD-MSCs. Expression of CD29 and CD44 had previously been investigated in AD-MSCs, BM-MSCs, and DP-MSCs; BM-MSCs showed higher expression of CD29 than DP-MSCs and AD-MSCs at the third passage [51]. These differences could reflect differences in the procedures used to isolate and cultivate MSCs and/or other biological factors. EGFP has been in routine use as a tag for in vivo model studies. It is considered that EGFP gene functionally is neutral when integrated and provides convenient cell labeling without a significant impact on basic cellular functions [52], even within MSCs [53]. We demonstrated that within our isolated MSCs, transduction of AD-MSCs, BM-MSCs, and DP-MSCs cells by LV-EGFP did not lead to significant changes in the expression of the investigated markers, which is consistent with other works [54].

The AD-MSCs applied after SCI in rats demonstrated the best results in restoring the motor activity (BBB score). However, it would be inaccurate to estimate the effectiveness of therapy based only on a behavioral test. In this regard, the summation of the data obtained from the M/H-responses, MEPs, and SEPs suggests the superiority of applying FM-embedded AD-MSCs compared to analogical application of FM-embedded MSCs obtained from other sources. Results obtained with BBB assessment are often deemed to be controversial as they do not account for variations in the underlying study design, for example, the variety of SCI models and their severity, differences in transplantation protocols (cell number, routes, and post-traumatic period of administration), the presence or absence of immunosuppression, and the quality of MSCs generated in culture. Given these caveats, a systematic review and meta-analysis of rat models of SCI demonstrates a difference in behavioral BBB locomotor score means of 3.9 in favor of treatment with MSCs [55].

In the present study, we found the restoration of the tissue integrity was improved with AD-MSCs treatment. The reduction of a total area of abnormal cavities and improved tissue retention were reported in numerous studies of MSC transplantation in SCI [56,57,58,59]. Ryu et al. [18] compared the efficiency of therapy of Matrigel mixed with MSCs derived from fat, bone marrow, Wharton’s jelly, and umbilical cord blood in dogs with spinal compression and similarly observed the lowest average lesion size in the adipose-derived MSCs group. MSCs can mediate anatomical improvement in the subacute phase after SCI through anti-inflammatory activity, glial scar reduction, and cell bridging effects [50,60]. Our results suggest that MSCs are able to prevent secondary injury by contributing to astroglia suppression, consistent with the results of other studies of MSC transplantation in SCI [61,62,63]. We observed greater reduction of astroglial activation caudally from the injury epicenter after AD-MSCs application compared to other sources of MSCs and the control groups. At the same time, we did not find a significant reduction of microglia pan marker Iba1 in the groups with MSCs transplantation.

Our findings within the rodent SCI model encouraged us to assess the effect of AD-MSCs in the subacute period of SCI in large animals. We evaluated the degree of injury and assessed the efficiency of AD-MSCs by the same criterions as in rats and we found similar results on the migration of MSCs after their application into the site of SCI. Our data also show tropism of transplanted AD-MSCs to the gray matter and their preferential migration through dorsal roots of the spinal cord that can be attributed to the presence of directional paths for their movement and the disrupted barrier in this area after SCI. The synechias (epidural fibrosis) was more pronounced in pigs compared to rats. In this regard, we also detected EGFP-labeled AD-MSCs in the area of epidural fibrosis, where significantly less MSCs were found in the synechias above the rostral part of spinal cord. These results may indicate the predominant migration of MSCs is in the rostral direction from the epicenter of SCI, which confirms our earlier in vitro model data (unpublished observations, [64]).

In contrast with the rodent model, we did not find a significant improvement in motor performance or increased behavioral scores in pigs that received treatment with AD-MSCs compared to the control group (FM application only). Park et al. [65] carried out intraspinal transplantation of MSCs derived from umbilical cord blood at 12 h and at 1 and 2 weeks after compression SCI in dogs; the animals receiving this therapy showed a significant increase in three different motor function scores after 1 week. However, application of a similar therapy, both in the acute (24 h) and delayed (2 weeks) period from SCI, did not lead to a motor function recovery. Similar outcomes were reported after intraspinal transplantation of Matrigel mixed with AD-MSCs, previously induced into neuronal differentiation at one week after SCI [66]. The electrophysiological changes observed in our study of pigs suggest that FM + AD-MSCs may have a positive effect on the restoration of long spinal cord tracts. These results are consistent with other studies that show improvement in SEPs in dogs and pigs with compression SCI after UCB-MSCs and BM-MSCs intraspinal injection [67,68]. Our results also suggest that application of AD-MSCs embedded in FM affects primarily distal to the injury site region of the spinal cord, although without significant restoration of conductivity across spinal tracts.

Similar to results in rats, application of AD-MSCs embedded in FM at the SCI site in pigs facilitated restoration of neural tissue integrity, as confirmed by morphometric analysis. The reductions of cavity formation and fibrosis after MSC transplantation in a large animal models of SCI were reported previously [65,66,67,68]. The identification of astroglial activation in pigs supports the capacity of AD-MSCs to reduce GFAP expression predominantly in the caudal direction from the epicenter of injury, similar to what we found in rats although to a lesser degree. The potential reduction in astroglial activation was attributed to the ability of MSCs to decrease cyclooxygenase-2 and IL-6 cytokine levels [18,59] and secretion of TNF-stimulated gene-6 [69,70,71,72], which consequently decreases NF-κB signaling, resulting in modulation of А1 neuroinflammatory reactive astrocytes [73,74]. We found no effect of AD-MSCs treatment on microglial activation in pigs, similar to results in rats. In addition, significant differences in Iba1^+^-microglia behavior in DREZ after SCI were found in both rats and pigs. In pigs, Iba1^+^-microglia in DREZ were mainly visualized as a compact cell clusters compared to pig intact control and in rats where the microglia were distributed more evenly. It was reported that clusters of activated microglia are formed mainly in the areas of active demyelination of white matter near the areas of active tissue damage in the brain of patients with multiple sclerosis and this specific microglia formation was not associated with destruction of blood–brain barrier [75]. Detected differences in microglia behavior in the area of SCI in rats and pigs have not previously been described and require further investigation.

## 5. Conclusions

The results of this study demonstrate that application of AD-MSCs combined with fibrin matrix during the subacute period in rats provides significantly higher post-traumatic regeneration compared to similar applications of BM-MSCs or DP-MSCs. Particularly, restoration of locomotor activity and conduction along spinal axons, reduction of post-traumatic cavitation, enhancing tissue retention, and modulation of microglial and astroglial activation were found in the rat model with AD-MSCs treatment. What mediates the greater therapeutic effect of AD-MSCs in SCI compared to BM-MSCs or DP-MSCs remains to be fully understood. However, biological advantages in the proliferative capacity, cell viability under oxidative stress, secreted proteins, and immunomodulatory effects have been previously discussed for AD-MSCs and may be critical factors [23,76]. These data indicate that application of MSCs combined with fibrin matrix into the surface of spinal cord may be an effective and safe approach for delivering cells to the damaged area. Furthermore, our results from pigs demonstrate partial replication of the findings observed in rats, regarding partial restoration of the somatosensory spinal pathways, reduction of post-traumatic cavitation, enhancing tissue retention, and modulation of astroglial activation in the DREZ.

Significant differences in functional recovery within the pig model could be attributed to the differences in the secretory phenotype of the transplanted cells and variable neuroregenerative potential of large animals. This is supported by significant differences in cytokine profile of MSCs obtained from different pigs; previous studies have also indicated donor-related heterogeneity of MSCs [77,78]. The therapeutic potential of adult neurogenesis is well recognized although the neuroregenerative capacity seems to be different between small and large animals and humans, which does not allow us to reach an optimal clinical outcome. The application of AD-MSCs embedded in FM at the site of SCI during the subacute period can stimulate important mechanisms of nervous tissue regeneration in both rats and pigs. Results in pigs confirm previous observations in rats and support the possible utility of AD-MSCs transplantation in large animals and in humans suffering subacute paraplegia. However, we need to more carefully analyze the secretory phenotype of donor cells, confirming how the therapeutic activity of transplanted MSCs is mediated before any possible clinical translation.

## Figures and Tables

**Table 1:** Primary and secondary antibodies used in flow cytometry and immunofluorescence staining.

Antibody	Host	Dilution	Source
Thy-1 (CD90) conjugated with РЕ/Cу5	Mouse	1:100	Вiolegend
CD 73	Rat	1:100	Вiolegend
CD 44 conjugated with APC/Cy7	Rat	1:100	Вiolegend
CD 29 conjugated with РЕ	Hamster	1:100	Вiolegend
CD34	Mouse	1:100	Santa Cruz
CD45	Rat	1:100	Sony
GFAP	Mouse	1:200	Santa Cruz
Iba1	Goat	1:300	Abcam
Anti-goat IgG conjugated with Alexa 555	Donkey	1:200	Invitrogen
Anti-mouse IgG conjugated with Alexa 546	Donkey	1:200	Invitrogen

**Table 2:** Flow cytometry data on the expression of surface antigens in cells derived from rat adipose tissue, bone marrow, and tooth pulp before/after transduction by LV-EGFP (%).

Source of MSC	Thy-1 (CD90)	CD73	CD44	CD29
Adipose tissue	99.9 ± 0.17/98.6 ± 2.3	94 ± 0.5/87.5 ± 4.4	98.4 ± 2.9/98.7 ± 2.6	88 ± 4/80 ± 8.5
Bone marrow	98.9 ± 1.6/96.8 ± 5.8	91 ± 0.5/88 ± 4	90.6 ± 5.8/93 ± 11.3	59 ± 7.3/51 ± 9 *
Dental pulp	91 ± 0.5/88.5 ± 1.2 *^#^	87.7 ± 1.7/87 ± 8	74 ± 0.5/69 ± 9 *^#^	49 ± 5.3/44 ± 0.5 *

* *p* < 0.05 as compared with analogically samples of AD-MSCs cultures, ^#^ *p* < 0.05 as compared with analogically samples of BM-MSCs cultures.

**Table 3:** Results of transcranial electrical stimulation in rats.

Groups of Animals	Amplitude of MEPs (mV)	Latency of MEPs (ms)	Lack of MEPs in Both Legs (in % of Rats)	Registration of MEPs from One Leg (in No. of Rats)
Intact control	17.66 ± 5.30	5.81 ± 0.63	-	-
FM	0.5 ± 0.36	15.83 ± 1.14	44%	1
FM + AD-MSCs + LV-EGFP	1.57 ± 0.88	14.08 ± 3.83	20%	1
FM + BM-MSCs + LV-EGFP	1.68 ± 0.7	14.77 ± 3.2	32%	-
FM + DP-MSCs + LV-EGFP	1.01 ± 0.75	12.85 ± 3.10	32%	-

**Table 4:** The AD-MSCs (left columns) and AD-MSCs + LV-EGFP (right columns) supernatant cytokine/chemokine concentrations (ng/mL).

Cytokine/ Chemokine	Pig 1	Pig 2	Pig 3	Pig 4	Pig 5
GM-CSF	<0.009/<0.009	<0.009/<0.009	<0.009/0.01 ± 0.0005	<0.009/<0.009	<0.009/<0.009
IFN-g	<0.12/<0.12	<0.12/<0.12	<0.12/<0.12	1.4 ± 0.1 */1.33 ± 0.15 *	1.2 ± 0.05 */<0.12
IL-1a	0.008 ± 0/0.009 ± 0.0005	0.008 ± 0/0.008 ± 0	0.009 ± 0/0.008 ± 0	0.009 ± 0.0005/0.008 ± 0	0.008 ± 0/0.008 ± 0
IL-1b	0.01 ± 0/0.01 ± 0	0.01 ± 0.0005/0.01 ± 0	0.01 ± 0/0.009 ± 0.001	0.009 ± 0/0.01 ± 0	0.009 ± 0.0005/0.01 ± 0
IL-1Ra	<0.03/<0.03	<0.03/<0.03	<0.03/<0.03	<0.03/<0.03	<0.03/<0.03
IL-2	<0.01/<0.01	<0.01/<0.01	<0.01/<0.01	<0.01/<0.01	<0.01/<0.01
IL-4	<0.05/<0.05	<0.05/<0.05	<0.05/0.05 ± 0.005	<0.05/0.03 ± 0.005	<0.05/<0.05
IL-6	2.77 ± 0.1 */2.5 ± 0.05 *	0.15 ± 0 */0.15 ± 0.01 *	9.21 ± 0.3 */9.4 ± 0.2 *	0.48 ± 0.01 */0.49 ± 0 *	0.57 ± 0.05 */0.62 ± 0 *
IL-8	21.05 ± 0.5 */20.5 ± 0.8 *	0.21 ± 0.01 */0.22 ± 0 *	14.55 ± 0.3 */15 ± 0.1 *	4.56 ± 0.2 */4.6 ± 0.05 *	3.02 ± 0.05 */3.2 ± 0.1 *
IL-10	<0.009/<0.009	<0.009/<0.009	<0.009/<0.009	0.01 ± 0.0005/<0.009	0.01 ± 0.0005/<0.009
IL-12	<0.03/<0.03	<0.03/<0.03	<0.03/0.03 ± 0.005	<0.03/0.03 ± 0.005	<0.03/<0.03
IL-18	0.1 ± 0 */0.1 ± 0 *	0.08 ± 0.001/0.09 ± 0	0.09 ± 0.001/0.09 ± 0	0.17 ± 0.02 */0.2 ± 0 *	0.35 ± 0 */0.4 ± 0.05 *
TNF-a	0.01 ± 0/0.01 ± 0	0.01 ± 0.0005/0.01 ± 0	0.01 ± 0/0.01 ± 0.005	0.01 ± 0/0.01 ± 0.005	0.01 ± 0.0005/0.01 ± 0

* *p* < 0.05 as compared with analogic samples of another pigs (AD-MSCs and AD-MSCs + LV-EGFP, accordingly). For values preceded with <, protein concentrations below the specified value cannot be detected with the kit.

## Supplementary Materials

The following are available online at https://www.mdpi.com/2218-273X/9/12/811/s1, Table S1: Primers and probes for RT-PCR, Video S1: Videotaping of locomotor recovery in the experimental groups in pigs.
