# Supplementary material for: Mesenchymal Stem Cell Therapy for Spinal Cord Contusion: A Comparative Study on Small and Large Animal Models
Source: Biomolecules. 2019 Dec 1;9(12):811. doi: 10.3390/biom9120811 (PMC6995633; doi:10.3390/biom9120811)
Supplement: Supplementary file 1 [file biomolecules-09-00811-s001.pdf]

Supplementary Information

Mesenchymal stem cells therapy for spinal cord contusion: comparative study on small and large animal models

Mukhamedshina et al.

Supplemental Table. Primers and probes for RT-PCR

Primer	Nucleotide sequence
18S-TM-Forward	gCCgCTAgAggTgAAATTCTTg
18S-TM-Reverse	CATTCTTggCAAATgCTTTCg
18S-TM-Probe	[HEX]ACCgCgCAAgACggACCAg [BH2]
V164-TM-Forward	TATATCTTCAAgCCgTCCTgTg
V164-TM-Reverse	TCTCCTATgTgCTggCTTTg
V164-TM-Probe	[FAM]TCCgCATgATCTgCATAgTgACgTTg [BH2]
Vim-TM-Forward	ACCCTgCAgTCATTCAgACA
Vim-TM-Reverse	TCCTggATCTCTTCATCgTg
Vim-TM-Probe	[HEX] CTggCACgTCTTgACCTTgAACg [BH2]
S100b-TM-Forward	GAgAgAgggTgACAAgCACA
S100b-TM-Reverse	CACCACTTCCTgCTCTTTgA
S100b-TM-Probe	[FAM] CgAgCTCTCTCACTTCCTggAggAA [BH1]
GFAP-TM-Forward	TTTCTCCAACCTCCAgATCC
GFAP-TM-Reverse	CTCCTTAATgACCTCgCCAT
GFAP-TM-Probe	[FAM] CCgCATCTCCACCgTCTTTACCA [BH1]
PDGFRa-TM-Forward	GgTTAgAggAgCACCTggAg
PDGFRa-TM-Reverse	TCTCACCTCACATCCgTCTC
PDGFRa -TM-Probe	[FAM] ATgCgCgACCTCCAACCTgA [BH1]
PDGFb-TM-Forward	CTgCAATAACCgCAATTgTg
PDGFb-TM-Reverse	TCgATCTTTCTCACCTgCAC
PDGFb-TM-Probe	[FAM] CCgCATCTgCACCTgCgAg [BH1]
FGF2-TM-Forward	GCTgCTggCTTCTAAgTgTg
FGF2-TM-Reverse	GTgCCACATACCAACTggAg
FGF2-TM-Probe	[FAM] TCTTCTTTgAACgCCTggAgTCCA [BH1]
HSPA1b-TM-Forward	CCAGGCAGGACCCAATCACA
HSPA1b-TM-Reverse	CGCAAGGTAGCGGTCTCTGT
HSPA1b-TM-Probe	(6-FAM)–CCGCCCAGCACTTTCAGGAGCTGACCC [BH1]
CNPase-TM-Forward	AGACATAGTGCCCGCAAAG
CNPase-TM-Reverse	GCTTGTCCTTAGCTCCTGAG
CNPase-TM-Probe	(6-FAM)–AGCCACACATTCCTGCCCAAGAT [BH1]
NGF-TM-Forward	CCAAGGACGCAGCTTTCTAT
NGF-TM-Reverse	CTCCGGTGAGTCCTGTTGAA

Iba1-TM-Forward	ACCAgCgTCTgAggAgCTAT
Iba1-TM-Reverse	AggAAgTgCTTgTTgATCCC
Iba1-TM-Probe	[HEX] CCCTgCAAATCCTTgCTCTggC [BH2]
mpz-TM-Forward	TCgCAAAgATgAgCAgAg
mpz-TM-Reverse	ggCCCATCATgTTCTTgA
mpz-TM-Probe	[FAM]CCAgTAGAACCAgCCTCAAgAAC [BH1]
Olig2-TM-Forward	AGTGCGCGATGCTAAGCTCT
Olig2-TM-Reverse	TGGGCCACGACACAGAAAGA
Olig2-TM-Probe	(6-FAM)–CGCGCCTCGTCGTCTAAGCCCGC [BH1]
Caspase3-TM-Forward	AATTCAAGGGACGGGTCATG
Caspase3-TM-Reverse	GCTTGTGCGCGTACAGTTTC
Caspase3-TM-Probe	[HEX] CggCCTCCACTggTATTTTATgACACg [BH2]
MBP-TM-Forward	ACACGGGCATCCTTGACTCCATCGG
MBP-TM-Reverse	TCCGGAACCAGGTGGTTTTCAGCG
GAP-43-TM-Forward	GCAGAAAAGAGGTGGAGAGG
GAP-43-TM-Reverse	TTGTTCAATCTTTTGGTCCTCATC
GAP-43-TM-Probe	[FAM] AGAGAAGGCAGGAAGAAGGCAGG [BH1]
